# Carnosic Acid Shows Higher Neuroprotective Efficiency than Edaravone or Ebselen in In Vitro Models of Neuronal Cell Damage

**DOI:** 10.3390/molecules29010119

**Published:** 2023-12-24

**Authors:** Danuta Jantas, Piotr Warszyński, Władysław Lasoń

**Affiliations:** 1 Maj Institute of Pharmacology, Polish Academy of Sciences, Department of Experimental Neuroendocrinology, 31-343 Krakow, Poland; lason@if-pan.krakow.pl; 2 Jerzy Haber Institute of Catalysis and Surface Chemistry, Polish Academy of Sciences, 30-239 Krakow, Poland; piotr.warszynski@ikifp.edu.pl

**Keywords:** hydrogen peroxide, glutamate, oxygen-glucose deprivation, primary cortical neurons, SH-SY5Y cells, ROS, MMP, caspase-3, excitotoxicity, neurotoxicity

## Abstract

This study compared the neuroprotective efficacy of three antioxidants—the plant-derived carnosic acid (CA), and two synthetic free radical scavengers: edaravone (ED) and ebselen (EB)—in in vitro models of neuronal cell damage. Results showed that CA protected mouse primary neuronal cell cultures against hydrogen peroxide-induced damage more efficiently than ED or EB. The neuroprotective effects of CA were associated with attenuation of reactive oxygen species level and increased mitochondrial membrane potential but not with a reduction in caspase-3 activity. None of the tested substances was protective against glutamate or oxygen-glucose deprivation-evoked neuronal cell damage, and EB even increased the detrimental effects of these insults. Further experiments using the human neuroblastoma SH-SY5Y cells showed that CA but not ED or EB attenuated the cell damage induced by hydrogen peroxide and that the composition of culture medium is the critical factor in evaluating neuroprotective effects in this model. Our data indicate that the neuroprotective potential of CA, ED, and EB may be revealed in vitro only under specific conditions, with their rather narrow micromolar concentrations, relevant cellular model, type of toxic agent, and exposure time. Nevertheless, of the three compounds tested, CA displayed the most consistent neuroprotective effects.

## 1. Introduction

Oxidative stress has long been recognized as the pivotal component of neuronal death in both acute (stroke, traumatic brain injury) and chronic neurodegenerative dis-eases, e.g., Alzheimer’s, Parkinson’s and Huntington’s disease [1,2,3]. It has been well established that oxidative stress results from a disturbed balance between the excessive intracellular accumulation of reactive oxygen species (ROS) and reactive nitrogen species (RNS) and endogenous antioxidant defense system in which glutathione peroxidase, glutathione reductase, superoxide dismutase, and catalase play the critical role [4]. The ROS and RNS in high concentrations are directly damaging factors for lipids, carbohydrates, amino acids, proteins and nucleic acids, in this way disrupting intracellular organelles, structural proteins and membranes [5,6]. Therefore, the removal of pathologically produced free radicals has been proposed as a viable neuroprotective strategy. Besides anti-oxidative enzymes, vitamins A, C and E, glutathione, plant polyphenolic compounds including flavonoids, thioredoxin, metallothionein, ceruloplasmin, and some trace elements can alleviate the harmful effects of ROS and RNS [2]. Although natural antioxidants show high activity in the scavenging of free radicals, their bioavailability is limited by low absorption and poor stability [7]. Regarding synthetic antioxidants, some compounds with strong free radical scavenging properties or free radical trapping activities (e.g., NXY-059—disufenton sodium and its derivatives) showed only modest neuroprotective activity and a bell-shaped dose–response curve in in vivo experimental models of neuronal damage. Moreover, in clinical trials, they failed to show consistent neuroprotective effects over placebo [8]. It should be mentioned here that clinical trials on the neuroprotective potential of antioxidants were conducted among small study populations [3]. On the other hand, some antioxidative compounds such as gallic acid esters, hydroxytoluene, and butylated hydroxyanisole display undesired effects on living organisms [9]. Among antioxidants with potential translational value, low molecular weight, and cell membrane-permeable superoxide dismutase mimetics, such as the nitroxide tempol (4-hydroxyl-2,2,6,6-tetramethylpiperidine-N-oxyl), seem quite promising [10]. The inconsistent results of studies on the neuroprotective effects of antioxidants are thought to be due to unfavorable pharmacokinetic profiles, i.e., low water solubility and bioavailability, difficult penetration through the blood–brain barrier (BBB), uncertain stability, and insufficient knowledge of their metabolism and elimination. Another problem concerns establishing therapeutic concentrations of antioxidants in blood and brain tissue because, depending on their concentrations, these compounds may exert antioxidative or prooxidative effects. One of the methods to improve the pharmacokinetic and pharmacodynamic properties of antioxidants is their encapsulation in nanoparticles (nanocarriers) [11,12]. However, before this step, it is essential to select the most promising antioxidant among various candidates in the same screening platforms for neuroprotection. 

**Figure 1:**
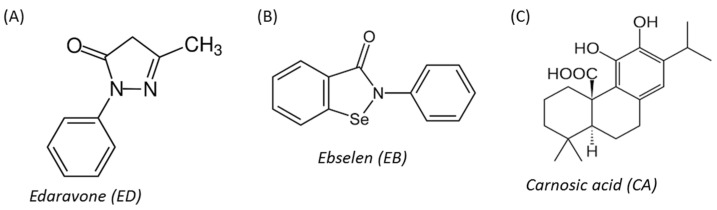
Chemical structure of edaravone (**A**), ebselen (**B**), and carnosic acid (**C**).

Based on the literature search, we have chosen three hydrophobic compounds with antioxidant properties: edaravone, ebselen, and carnosic acid. Edaravone (ED, MCI-186, 3-methyl-1-phenyl-2-pyrazolin-5-one, Figure 1A) is a clinical drug developed by Mitsubishi Tanaba (Osaka, Japan) and has been approved by Japan and the FDA for ALS treatment since 2015 and 2017, respectively [13]. It is a free radical scavenger with the capacity to mitigate oxidative injury in various models of neuronal damage. The protective effects of ED in attenuating NO, glutamate, and hypoxia-induced cytotoxicity and apoptosis have been reported [14,15,16,17]. ED also effectively protects astrocytes from oxidative stress or infectious insults such as bacterial lipopolysaccharides [18]. Ebselen (EB, 2-phenyl-1,2-benzisoselenazol-3(2H)-one, Figure 1B) is an organoselenium compound with well-characterized toxicology and pharmacology [19]. Its antioxidative mechanism of action involves glutathione peroxidase-like activity and ability to react with thiols, peroxynitrites, and hydroperoxides. EB protects cell components from oxidative damage [20,21]. EB and its analogues showed neuroprotective effects in various experimental models against cell damage induced by oxygen and glucose deprivation (OGD), amyloid β(1-42), lipopolysaccharide, 6-hydroxydopamine (6-OHDA), and in MPTP-treated mice [22,23,24,25,26]. Carnosic acid (CA, 4aR,10aS)-5,6-dihydroxy-7-isopropyl-1,1-dimethyl-1,3,4,9,10,10a-hexahydro-2H-phenanthrene-4a-carboxylic acid, Figure 1C) isolated from rosemary (Rosmarinus officinalis) and common sage (Salvia officinalis) possesses antioxidative, anti-inflammatory, and anti-neoplastic properties [27,28,29]. CA was found to ameliorate oxidative stress-, glutamate-, and hypoxia-induced injury of neuronal as well as displayed neuroprotective activity in in vitro and in vivo models of Parkinson’s or Alzheimer’s disease [30,31,32,33,34,35,36,37,38,39]. 

Although most of the above-cited studies unanimously indicate the neuroprotective effects of ED, EB, and CA, they differ in experimental settings, doses of compounds, times of exposures, and measurements of cellular damages, etc., which makes their comparison difficult. Therefore, in order to select the most promising neuroprotective compound of those three for nanoencapsulation for future experimental studies, it was necessary to estimate their properties under similar, well-controlled conditions. Thus, in the present study, we compared biocompatibility and neuroprotective potentials of ED, EB, and CA in a wide range of concentrations in mouse primary neuronal cell cultures exposed to oxidative stress inducer (hydrogen peroxide, H_2_O_2_), excitotoxic factor (glutamate), and OGD. Moreover, some protective mechanisms were studied for the best-acting neuroprotectant. Finally, biosafety and neuroprotective profiles of these three compounds were also tested in the human neuronal-like model: undifferentiated (UN-) and retinoic acid-differentiated (RA-) neuroblastoma SH-SY5Y cells exposed to H_2_O_2_.

## 2. Results and Discussion

### 2.1. The Effect of Edaravone in Primary Neuronal Cell Cultures

**Figure 2:**
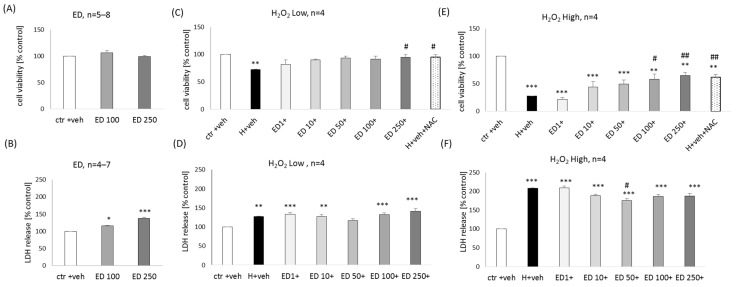
Biosafety (**A**,**B**) and neuroprotection (**C**–**F**) assessment against the hydrogen peroxide (H_2_O_2_)-induced cell damage by edaravone (ED) in primary neuronal cell cultures. The eight days in vitro cortical neurons were treated either with vehicle or with ED alone (100 and 250 μM) or ED (1–250 μM) in combination with low (150 μM) or high (200 μM) concentrations of H_2_O_2_ for 24 h. An antioxidant N-acetyl-cysteine (NAC, 1 mM) was used as a positive control of the model. Cell viability (**A**,**C**,**E**) and cytotoxicity (**B**,**D**,**F**) were measured by MTT reduction and LDH release assays, respectively. The data were normalized to vehicle-treated cells and presented as the mean ± SEM. The number of independent experiments (*n*) is indicated in each graph. * *p* < 0.05, ** *p* < 0.01 and *** *p* < 0.001 vs. vehicle-treated cells; # *p* < 0.05 and ## *p* < 0.01 vs. H_2_O_2_-treated cells.

ED at concentrations of 100 and 250 μM did not evoke any reduction in cell viability in primary neuronal cell cultures (Figure 2A) but slightly increased the LDH release (17–37%) (Figure 2B). A significant neuroprotective effect of ED (100 and 250 μM) was found in the model of neuronal cell damage induced by lower (150 μM) and higher (200 μM) concentrations of H_2_O_2_ at the level of the cell viability assessment. This effect was comparable to protection mediated by positive control, NAC (1 mM) (99.28% and 94.15–105.29% of NAC efficiency for low and high H_2_O_2_, respectively) (Figure 2C,E). In the cytotoxicity assay, a slight reduction was observed of the high H_2_O_2_-evoked changes in this parameter by ED at a concentration of 50 μM (Figure 2F), but no impact of ED was found on low H_2_O_2_-induced LDH release (Figure 2D). 

In excitotoxicity (Glu) and OGD models of neuronal cell damage, we did not notice any protection mediated by ED (1–250 μM) in both MTT reduction and LDH release assays (Figures S1A,B and S2A,B). However, a protective response of positive control, MK-801 (1 μM) was confirmed in each model and assay (Figures S1A,B and S2A,B).

### 2.2. The Effect of Ebselen in Primary Neuronal Cell Cultures

**Figure 3:**
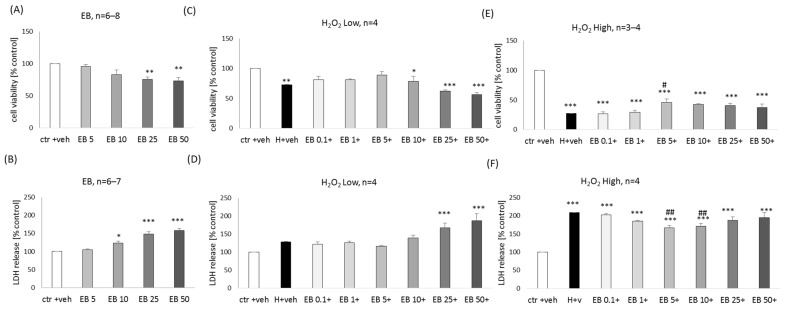
Biosafety (**A**,**B**) and neuroprotection (**C**–**F**) assessment against the hydrogen peroxide (H_2_O_2_)-induced cell damage by ebselen (EB) in primary neuronal cell cultures. The eight days in vitro cortical neurons were treated either with vehicle or with EB alone (5–50 μM) or EB (0.1–50 μM) in combination with low (150 μM) or high (200 μM) concentrations of H_2_O_2_ for 24 h. Cell viability (**A**,**C**,**E**) and cytotoxicity (**B**,**D**,**F**) were measured by MTT reduction and LDH release assays, respectively. The data were normalized to vehicle-treated cells and presented as the mean ± SEM. The number of independent experiments (*n*) is indicated in each graph. * *p* < 0.05, ** *p* < 0.01 and *** *p* < 0.001 vs. vehicle-treated cells; # *p* < 0.05 and ## *p* < 0.01 vs. H_2_O_2_-treated cells.

In primary neuronal cell cultures, EB at concentrations of 25 and 50 μM evoked a significant reduction (about 25%) in cell viability (Figure 3A), and at concentrations of 10–50 μM, gradually increased (23–57%) LDH release (Figure 3B). A moderate neuroprotective effect of EB was found only in the model of cell damage induced by high H_2_O_2_ (73.56% of NAC efficiency), and it was significant for the concentration of 5 μM in MTT reduction assay (Figure 3E) and for concentrations 5 and 10 μM in LDH release assay (Figure 3F). 

In the Glu model of neuronal cell damage, we did not notice any protection mediated by EB (0.1–50 μM), but a significant increase in the extent of cell damage (10–30%) was found for all tested concentrations of EB in MTT reduction assay (Figure S1C) and for its higher concentrations (25 and 50 μM, about 22–25%) also in LDH release assay (Figure S1D). In the OGD model of neuronal cell damage, we also did not find any protection mediated by EB (1–10 μM) in both MTT reduction and LDH release assays (Figure S2C,D). However, in the cell viability assay, we found an exaggeration of the OGD-evoked cell damage by 20% by 10 μM EB (Figure S2C). 

### 2.3. The Effect of Carnosic Acid in Primary Neuronal Cell Cultures

**Figure 4:**
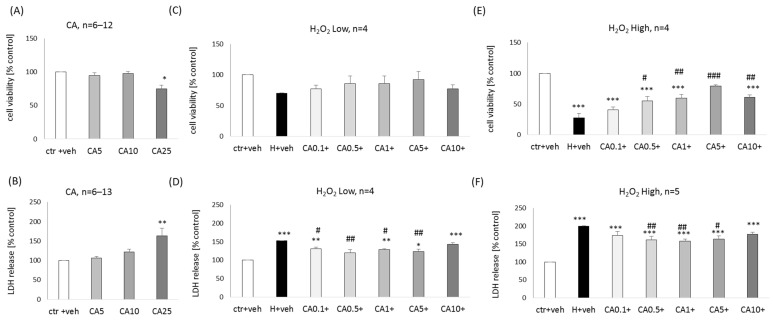
Biosafety (**A**,**B**) and neuroprotection (**C**–**F**) assessment against the hydrogen peroxide (H_2_O_2_)-induced cell damage by carnosic acid (CA) in primary neuronal cell cultures. The eight days in vitro cortical neurons were treated either with vehicle or with CA alone (5–25 μM) or CA (0.1–10 μM) in combination with low (150 μM) or high (200 μM) concentrations of H_2_O_2_ for 24 h. Cell viability (**A**,**C**,**E**) and cytotoxicity (**B**,**D**,**F**) were measured by MTT reduction and LDH release assays, respectively. The data were normalized to vehicle-treated cells and presented as the mean ± SEM. The number of independent experiments (*n*) is indicated in each graph. * *p* < 0.05, ** *p* < 0.01 and *** *p* < 0.001 vs. vehicle-treated cells; # *p* < 0.05, ## *p* < 0.01 and ### *p* < 0.001 vs. H_2_O_2_-treated cells.

In primary neuronal cell cultures, CA at a concentration of 25 μM evoked a significant reduction (about 25%) in cell viability (Figure 4A) and increase (about 60%) in LDH release (Figure 4B). In the low H_2_O_2_-evoked cell damage model, we did not find protection by CA at the level of cell viability measurement (Figure 4C), but in cytotoxicity assay, we noted partial reduction (by about 22–33%) of the H_2_O_2_-stimulated LDH release by CA at concentrations 0.1–5 μM (Figure 4D). In the high H_2_O_2_-evoked cell damage model, a significant increase (28–52%) in cell viability (Figure 4E) and reduction (by about 40%) of LDH release (Figure 4F) was found for by CA at concentrations 0.5–10 μM and 0.5–5 μM, respectively. The protection mediated by CA in cell viability assay ranged from 89.12% of NAC efficiency for 0.05 μM CA up to 128.27% of NAC efficiency for 5 μM CA. 

**Figure 5:**
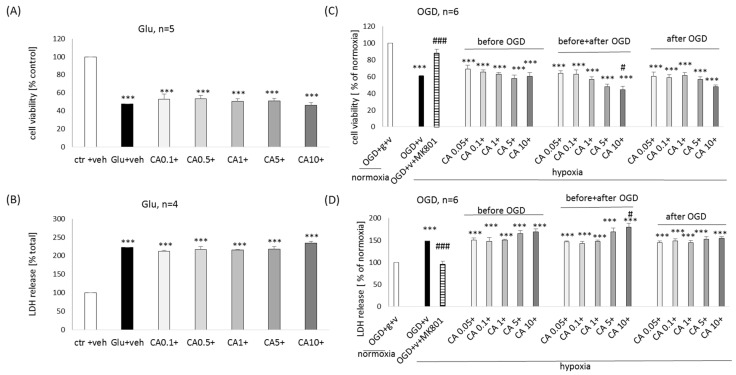
Neuroprotective effect of carnosic acid (CA) against the glutamate (Glu, **A**,**B**)- or oxygen-glucose deprivation (OGD, **C**,**D**)-induced cell damage in primary neuronal cell cultures. (**A**,**B**) The eight days in vitro cortical neurons were treated either with vehicle or with CA (0.1–10 μM) in combination with Glu (1 mM) for 24 h. (**C**,**D**) The eight days in vitro cortical neurons were treated either with vehicle or with CA (0.05–10 μM) under three schedules (before OGD, before + after OGD, after OGD) combined with a 3 h OGD procedure and 24 h of reoxygenation period. NMDA receptor antagonist MK-801 (1 μM) was used as a positive control to the model. Cell viability (**A**,**C**) and cytotoxicity (**B**,**D**) were measured by MTT reduction and LDH release assays, respectively. The data were normalized to vehicle-treated cells and presented as the mean ± SEM. The number of independent experiments (*n*) is indicated in each graph. *** *p* < 0.001 vs. vehicle-treated cells; # *p* < 0.05 and ### *p* < 0.001 vs. OGD-treated cells.

In the Glu model of neuronal cell damage, we did not notice any protection mediated by CA (0.1–10 μM) in both MTT reduction (Figure 5A) and LDH release (Figure 5B) assays. In the OGD model of neuronal cell damage, we also did not find any protection mediated by CA (1–10 μM) in both assays for assessment of cell viability/toxicity (Figure 5C,D). Moreover, in the MTT assay for CA at a concentration of 10 μM (given before and after OGD), we found a significant exaggeration (about 16%) of the OGD-evoked cell damage (Figure 5C). 

### 2.4. Mechanisms of CA-Mediated Neuroprotection in Primary Neuronal Cell Cultures

**Figure 6:**
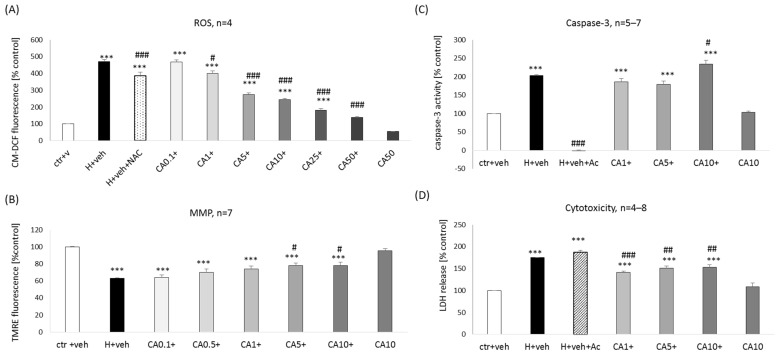
Effect of carnosic acid (CA) on the H_2_O_2_-evoked changes in reactive oxygen species (ROS, **A**) level, mitochondrial membrane potential (MMP, **B**), caspase-3 activity (**C**), and cytotoxicity (**D**) in primary neuronal cell cultures. (**A**) ROS production was assayed by loading the eight days in vitro cortical neurons with 5 μM of CM-H2DFFDA followed by treatment with CA (0.1–50 μM), antioxidant N-acetylcysteine (NAC, 1 mM), and H_2_O_2_ (1 mM) for 1 h. (**B**) For MMP assessment, the eight days in vitro cortical neurons were treated with CA (0.1–10 μM) and H_2_O_2_ (200 μM) for 6 h. After treatment, the cells were loaded with TMRE (100 nM) for 30 min. (**C**,**D**) For caspase-3 activity and cytotoxicity measurements, the eight days in vitro cortical neurons were treated with CA (1–10 μM) and H_2_O_2_ (200 μM) for 9 h. A caspase-3 inhibitor, Ac-DEVD-CHO (Ac, 20 μM) was used as a positive control to the assay. After treatment, caspase-3 activity (**C**) in cells was measured using fluorogenic substrate Ac-DEVD-AMC, and in the cell culture medium, LDH level (**D**) was measured. Fluorescence (**A**–**C**) or absorbance (**D**) of all probes were determined using multi-well plate reader. Data after normalization to vehicle-treated cells (100%) are presented as a mean ± SEM. The number of independent experiments (n) is indicated in each graph. *** *p* < 0.001 vs. vehicle-treated cells; # *p* < 0.05, ## *p* < 0.01 and ### *p* < 0.001 vs. H_2_O_2_- treated cells.

Since neuroprotection of tested compounds was demonstrated only in the model of cell damage induced by oxidative stress inducer H_2_O_2_, we first verified the impact of the best-acting neuroprotectant (CA) on intracellular ROS production. We found that CA (0.1–50 μM) in a concentration-dependent manner decreased the H_2_O_2_-stimulated ROS production (Figure 6A), and this effect was higher than one mediated by antioxidant NAC (1 mM). CA (50 μM) alone did not affect the intracellular ROS level (Figure 6A).

One of the mechanisms of cell damage induced by H_2_O_2_ is its detrimental impact on mitochondria, leading to a decrease in mitochondrial membrane potential (MMP) [40]. We demonstrated that after 6 h exposure to H_2_O_2_ (200 μM), there was about a 40% decrease in MMP, which was partially alleviated by CA at concentrations 5 and 10 μM (Figure 6B).

A decrease in MMP could lead to the release of pro-apoptotic factors like cytochrome c or AIF (apoptosis-inducing factor), which in the next steps could activate caspase 3-dependent or caspase-3-independent apoptosis, respectively [41]). Thus, we verified if CA has any effect on H_2_O_2_-induced caspase-3 activity. We showed an almost two-fold increase in caspase-3 activity after 9 h of treatment with H_2_O_2_ (200 μM), which was completely inhibited by caspase-3 inhibitor, Ac-DVD-CHO (20 μM) but not affected by CA (1–5 μM) (Figure 6C). In parallel, we observed that CA (1–10 μM) attenuated the H_2_O_2_-evoked LDH release (Figure 6D), evidencing shorter than 24 h exposure protective action of CA. Although CA, at a concentration of 10 μM slightly increased the H_2_O_2_-induced caspase-3 activity (Figure 6C), it did not have any detrimental effect since, in the cytotoxicity test, we observed protection by CA at this concentration (Figure 6D).

**Figure 7:**
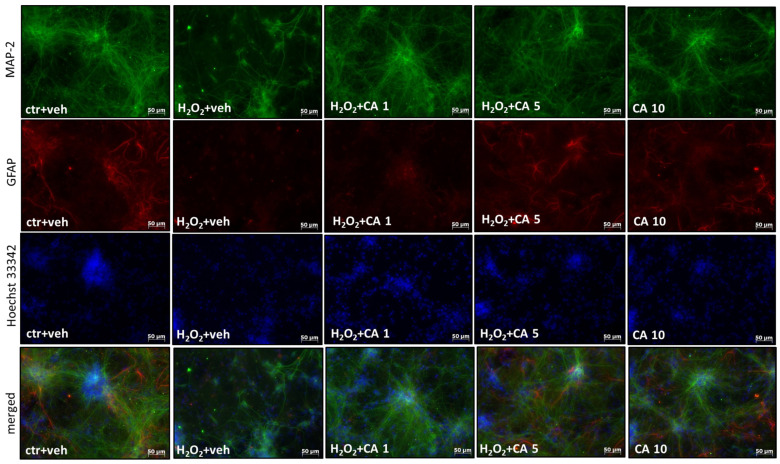
Representative microphotographs of primary neurons treated with carnosic acid (CA, 1–10 μM) and H_2_O_2_ (200 μM) for 24 h. After the treatment, the cortical neurons were fixed and immunostained with a neuronal marker (anti-MAP-2, green), astrocyte marker (anti-GFAP, red), and nuclear dye Hoechst 33342 (blue).

At the level of morphological changes visualized by immunofluorescent staining of neuronal (MAP-2) and glia (GFAP) cells, we observed that after 24 h exposure of primary neuronal cell cultures to H_2_O_2_ (200 μM), there was a reduction in the number of neuronal cells and complete demise of astrocytes (Figure 7). In parallel, in Hoechest 33342 staining, we observed after H_2_O_2_ treatment an increase in the number of pyknotic nuclei, which is the hallmark of condensed or fragmented DNA evoked by apoptotic- and necrotic-like processes (Figure 7). CA at concentrations 1 and 5 μM prevented the H_2_O_2_-evoked detrimental changes in neuronal cells, and at a concentration of 5 μM also protected glia cells (Figure 7). 

### 2.5. Influence of the Type of Experimental Medium on Biosafety of ED, EB, and CA in Human Neuroblastoma SH-SY5Y Cells 

**Figure 8:**
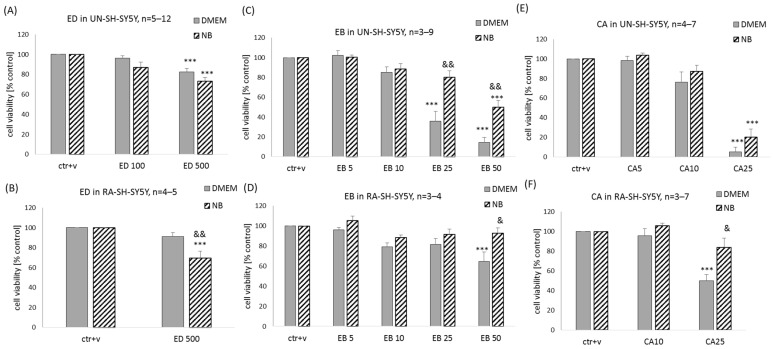
The impact of various experimental mediums (DMEM vs. NB) on the biosafety profile of edaravone (ED, (**A**,**B**)), ebselen (EB, (**C**,**D**)), and carnosic acid (CA, (**E**,**F**)) in undifferentiated (UN-) and retinoic acid (RA-)-differentiated SH-SY5Y cells. The UN- and RA-SH-SY5Y cells growing overnight in neuroblastoma (DMEM) or neuronal (NB) experimental medium were treated with ED (100 and 500 μM), EB (5–50 μM), or CA (5–25 μM) for 24 h. Cell viability was measured by WST-1 assay. Data after normalization to vehicle-treated cells (100%) are presented as a mean ± SEM. The number of independent experiments (*n*) is indicated in each graph. *** *p* < 0.001 vs. vehicle-treated cells; ^&^ *p* < 0.05 and ^&&^ *p* < 0.01 NB vs. DMEM medium for indicated drug concentration.

We tested the impact of neuroblastoma experimental medium (DMEM) and neuronal one (NB) on potential detrimental effects of ED, EB, and CA in two SH-SY5Y cell phenotypes, UN- and RA-SH-SY5Y. We demonstrated with WST-1 cell viability assay that after 24 h of treatment with ED at a concentration of 500 μM, there was about 20–25% reduction in cell viability in UN-SH-SY5Y cells, and this effect was not statistically different between cells cultured in DMEM and NB (Figure 8A). In RA-SH-SY5Y, we observed significant cell damage evoked by 500 μM ED only in cells cultured in NB, and it was statistically different to cells cultured in DMEM (Figure 8B). However, additional statistical analysis by two-way ANOVA to compare UN- vs. RA-SH-SY5Y cells in particular experimental medium type did not reveal the significant impact of cell phenotype on the cell-damaging effect of ED (500 μM) (*p* = 0.151 and *p* = 0.620 for DMEM and NB, respectively). 

In the case of EB, we noticed a higher cell-damaging effect in UN-SH-SY5Y cells when compared to RA-SH-SY5Y cells, and this effect was also dependent on the type of experimental medium. The highest detrimental effect of EB was observed for concentrations 25 and 50 μM in UN-SH-SY5Y cultured in DMEM (reduction in cell viability by 65 and 85%, respectively) when in NB medium, cell damage was evoked by EB only at concentration 50 μM (reduction by 50%) (Figure 8C). In the case of RA-SH-SY5Y cells, there was a significant reduction in cell viability by 50 μM EB in cells cultured in DMEM but not in NB (Figure 8D). Additional statistical analysis by two-way ANOVA to compare UN- vs. RA-SH-SY5Y cells in particular experimental medium type revealed a significantly higher resistance of RA-SH-SY5Y cells to the cell-damaging effect of EB (*p* = 0.00009 and *p* = 0.00266 for DMEM and NB, respectively). 

We noticed that CA at a concentration of 25 μM but not at 10 μM evoked a similar reduction of cell viability (by about 90%) in UN-SH-SY5Y cells cultured in DMEM and NB (Figure 8E). In RA-SH-SY5Y, we observed significant cell damage evoked by 25 μM CA only in cells cultured in DMEM (reduction by 50%). It was statistically different to cells cultured in NB, where this concentration of CA was not statistically different from control cells (Figure 8F). Moreover, additional statistical analysis by two-way ANOVA to compare UN- vs. RA-SH-SY5Y cells in particular experimental medium type revealed a significantly higher resistance of RA-SH-SY5Y cells to the cell-damaging effect of CA (*p* = 0.00071 and *p* = 0.00001 for DMEM or NB, respectively). 

### 2.6. Influence of the Type of Experimental Medium on Neuroprotective Potential of ED, EB, and CA in Human Neuroblastoma SH-SY5Y Cells against the H_2_O_2_-Evoked Cell Damage

We observed higher toxicity of H_2_O_2_ (375 μM) in UN-SH-SY5Y cells cultured in NB compared to DMEM (reduction in cell viability by 80 and 40%, respectively) (Figure S3A,B). The RA-SH-SY5Y cells were more resistant to H_2_O_2_-evoked cell damage when compared to UN-SH-SY5Y, as evidenced by the use of a higher concentration of H_2_O_2_ (500 μM). The impact of the experimental medium on the detrimental effect of this oxidative stress inducer was also observed, higher in NB when compared to DMEM (reduction in cell viability by 70 and 30%, respectively). These observations were also confirmed by the LDH release assay (Table 1). 

Twenty-four hours of co-treatment of UN- or RA-SH-SY5Y cells with ED (1–250 μM) did not attenuate the extent of cell damage evoked by H_2_O_2_ in any type of the used experimental medium (DMEM or NB), whereas NAC (1 mM) was protective in all conditions (Figure S3). We also found no protection by EB (0.1–50 μM) against the H_2_O_2_-evoked detrimental effects in UN- and RA-SH-SY5Y cells under various experimental settings (Figure S4). Moreover, in UN- and RA-SH-SY5Y cells cultured in DMEM, we observed potentiation in the H_2_O_2_-cell damaging effect by 50 μM EB (Figure S4A,C).

**Figure 9:**
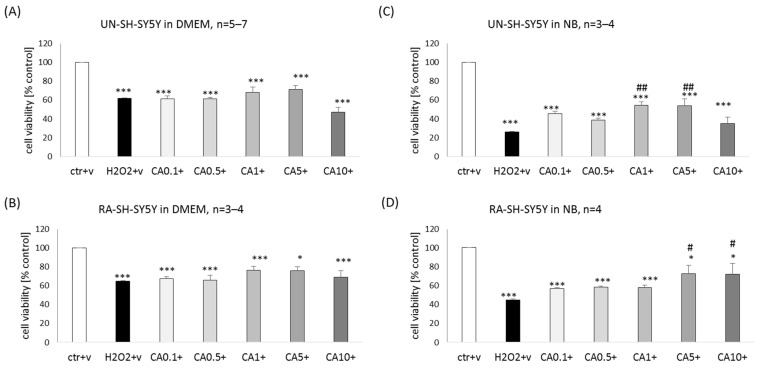
Neuroprotective effect of carnosic acid (CA) against the H_2_O_2_-induced cell damage in UN- and RA-SH-SY5Y under various experimental mediums (DMEM vs. NB). The UN- and RA-SH-SY5Y cells growing overnight in neuroblastoma (DMEM), or neuronal (NB) experimental medium were treated either with vehicle or with CA (0.1–10 μM) in combination with H_2_O_2_ (375 and 500 μM for UN- and RA-SH-SY5Y cells, respectively) for 24 h. Cell viability was measured by WST-1 assay. Data after normalization to vehicle-treated cells (100%) are presented as a mean ± SEM. The number of independent experiments (*n*) is indicated in each graph. * *p* < 0.05 and *** *p* < 0.001 vs. vehicle-treated cells; # *p* < 0.05 and ## *p* < 0.01 vs. H_2_O_2_- treated cells.

Twenty-four hours’ co-treatment of UN-SH-SY5Y cells with CA (0.1–10 μM) in DMEM experimental medium did not attenuate the extent of cell damage evoked by H_2_O_2_ (Figure 9A,C). However, in the cytotoxicity assay, we found a significant attenuation of the H_2_O_2_-induced LDH release by CA 1 and 5 μM (Table 1). We did not notice any protection by CA (0.1–10 μM) in RA-SH-SY5Y cells cultured in DMEM in both WST-1 (Figure 9C) and LDH release (Table 1) assays. There was significant protection mediated by CA in UN- and RA-SH-SY5Y cells cultured in NB experimental medium for concentrations 1–5 μM and 5–10 μM for UN- and RA-SH-SY5Y, respectively (Figure 9B,D). Additionally, we confirmed these results by an LDH release assay (Table 1). It should be noted that in cell viability assay in UN-SH-SY5Y cells, the extent of protection mediated by CA (1 and 5 μM) was comparable to the effect of NAC (about 95% of NAC efficiency), whereas in RA-SH-SY was higher than NAC effects (about 128% of NAC efficiency).

Neuroprotective effects of natural and synthetic antioxidants such as ED, EB, and CA have been widely documented, and the intracellular mechanisms of their action are rather well recognized [20,24,26,27,28,42,43]. However, our comparative in vitro study using various neuronal cell models and experimental settings only to some extent supported the general view on the biosafety of these compounds as well as the high efficacy of them in protecting neuronal cells against various harmful insults. 

When considering the biosafety profile of tested three antioxidants (ED, EB, and CA), we showed that incubation of primary cortical neuronal cells with EB or CA in higher concentrations (25 and 50 μM) decreased the viability of these cells. Cytotoxic effects of EB were also found by Zhang et al. [44], who showed that EB at concentrations above 10 μM for 24 h induced cell damage in human multiple myeloma cell lines by enhancing the production of endogenous ROS and triggering mitochondria-mediated apoptotic pathway. Similar cell-damaging effects of EB were demonstrated by other research groups, for example, in rat C6 glioma cells, human glioma cell lines (U87-MG, A172 or T98G) or in lung cancer cell lines (A549, Calu-6) [44,45,46,47]. Our data demonstrates that EB up 10 μM was safe for neuronal cells are in line with results obtained by other research groups in primary hippocampal of cerebellar granule cell cultures [23,48] and in rat hippocampal slices [26]. CA is considered a relatively safe compound (up to 10 µM) [27,28,49] as was confirmed by our results from primary neurons or SH-SY5Y cells.

The cytotoxic effects of CA were observed by us in neuronal cells only at higher concentrations (above 25 μM), which is in line with findings from rat PC12 cells [50]. It should be added that CA at higher concentrations (above 20 μM) demonstrated cell-damaging activity in various cancer cell lines and was postulated as a promising anticancer compound [29]. Interestingly, in SH-SY5Y cells, which have a tumor origin, we showed a higher cytotoxic effect of EB and CA in undifferentiated cells when compared to RA-differentiated. Moreover, we noticed in both cell phenotypes that culturing the cells in DMEM medium reveals a higher cell-damaging effect of EB than in NB one. In the case of CA, such association was observed only in RA-SH-SY5Y, since in UN-SH-SY5Y cells we observed the same extent of cell death evoked by CA (25 μM) in DMEM and NB. The observed cytotoxic effects of EB and CA in high concentrations might be explained by their prooxidative properties, as was reported previously [44,47,51,52,53]. It is not excluded that components of the DMEM medium favor this effect more than those present in the NB medium; however, this phenomenon needs a detailed experimental exploration.

Moreover, in the present study, we confirmed that the third investigated compound ED in concentration up to 250 μM was safe for neuronal cells, as was evidenced by cell viability assays and by morphological observations, which was also reported previously in various neuronal and non-neuronal cell types [18,54,55,56,57]. However, the noted increased level of LDH released to the medium after ED (100 and 250 μM) exposure could be explained by its nonspecific biochemical interaction with the assay rather than biological response (cytotoxic activity). Thus, caution should be kept when using various biochemical assessments which could be affected by some compounds. Moreover, our study performed with SH-SY5Y cells under differential cell culture conditions showed that ED at a concentration of 500 μM could evoke some reduction in cell viability in undifferentiated but not in RA-differentiated cells cultured in the DMEM experimental medium. Interestingly, placing the cells in the NB experimental medium did not change the cell-damaging effect of ED in UN-SH-SY5Y when compared to the DMEM medium. However, this detrimental effect was also observed in RA-SH-SY5Y cells when cultured in NB medium. Thus, components of NB medium did not protect against potential ED cytotoxic effects, whereas DMEM medium did, which is also an intriguing effect which needs more detailed evaluation. Nevertheless, to our knowledge, such comparative studies on the impact of various types of the experimental medium have not been conducted previously, and we point to it as an important factor which could significantly affect the biosafety profile of tested compounds. Hence, further studies on the neuroprotective potency of EB and CA were performed using lower concentrations of these compounds.

When it comes to neuroprotective potency assessment of evaluated compounds, we demonstrated that ED and EB only moderately protected primary neuronal cells in a rather narrow range of their concentrations and only against the H_2_O_2_-induced damage, while they were inactive or even slightly enhanced (EB) the Glu or the OGD detrimental effects. In contrast to them, an NMDA receptor antagonist, MK-801, significantly attenuated both the Glu and the OGD neurotoxicity, which positively verifies our experimental models. Our data from EB effects in the Glu model of neuronal cell damage are in contrast to results obtained by Xu et al. [58], who showed that EB at concentrations 4–12 μM, but not at lower or higher ones, attenuated the cell damage induced by Glu in mouse primary 6–7 days cortical neurons. This effect was associated with the inhibition of apoptotic changes evoked by Glu and the regulation of Bcl-2 and Bax proteins. It is not excluded that the differences in cell culture medium composition and discrepancies in protocols for Glu exposure) between Xu et al. [58] (DMEM medium containing 10% fetal bovine serum, 50 μg/mL gentamicin, and 25 mM KCl; 1 mM Glu for 20 min. in Locke’s solution followed by 12 h in experimental medium) and our (Neurobasal_A medium, B27 supplement without antioxidants, 2 mM L-glutamine and penicillin (0.06 μg/mL)/streptomycin (0.1 μg/mL) solution); 1 mM Glu in experimental medium for 24 h) study, are responsible for observed protection by EB in the former study and lack of protection in the latter one. The enhancing effect of EB on the OGD-induced neuronal damage is in line with the previous report by Shi et al. [47], who found that this compound at concentration 5–20 μM further increased C6 glioma cell death in the OGD model and suggested that EB can have a beneficial or toxic effect, depending on the availability of GSH. However, in undifferentiated SH-SY5Y cells exposed to the OGD/reoxygenation model, Landgraft et al. [24] demonstrated the protective effect of EB or its analogues at a concentration 10 μM but not higher (20 or 30 μM). Porciúncula et al. [26] also showed that EB (1 and 10 μM) was protective against the OGD-evoked damage in hippocampal slices only when added before and after the OGD procedure, not when administrated 30 min after OGD. In our study, we did not find any protection by EB against the OGD-evoked cell death under any of the administration procedures (before, before and after, or after OGD), which suggests that the protective effects of EB against OGD could be cell type-specific. A similar assumption could be applied to the H_2_O_2_ model since, in our study, we observed some protection by EB (5 and 10 μM) against the H_2_O_2_-evoked cell damage in primary neurons but not in SH-SY5Y cells cultured in various experimental medium (DMEM and NB). The latter is consistent with results from Wedding et al. [21], who failed to demonstrate any protection by EB (10 μM) against the H_2_O_2_-induced cell death in undifferentiated SH-SY5Y cells. However, Yoshizumi et al. [59] showed that EB (10 and 100 μM) attenuated the PC12 cell damage induced by H_2_O_2_, which was associated with the inhibition of the JNK and AP-1 signaling pathway. 

The observed lack of effect in the present study of ED on the OGD-induced damage of primary cortical neurons is in contrast to data by Song et al. [55], who found that ED (0.01–1 μM) protected rat PC12 cells from apoptosis and necrosis evoked by OGD/reoxygenation injury. The reason for this discrepancy may be due to the different cell types used in our and Song’s experimental models. The same explanation could be applied to the discussion of our results with Bai et al. [14], who showed that ED (250–750 μM) protected spiral ganglion neurons against the Glu-evoked excitotoxicity, whereas we did not find any effect of ED in a similar model in cortical neurons. Zhao et al. [57] also noted the protective effects of ED (10–100 μM) against the Glu-evoked cell damage in mouse hippocampal HT-22 cells, but in this case, when cells with glycolytic phenotype were used, it was rather cell damage executed by oxytosis (oxidative stress-induced cell damage). Wu et al. [56] also showed protection by ED (100–200 μM) against the Glu-evoked excitotoxicity in 7 days in vitro rat hippocampal neurons, which is in contrast to our data from primary cortical neurons, but they found protection against the H_2_O_2_ model, which is in line with our findings. The discrepancies between Wu et al. and our results regarding the Glu model could be explained by different species, brain region of cell origin, and differential experimental settings. In the former study, hippocampal neurons originated from postnatal day one rat pups and were cultured during the experiment in Neurobasal medium containing phenol red, B27 supplement, 0.5 mM L-glutamine, and penicillin (100 units/mL)/streptomycin (100 μg/mL) solution. In our study, cortical neurons originated from embryonic day 15 and were cultured in Neurobasal_A medium, B27 supplement without antioxidants, 2 mM L-glutamine, and penicillin (0.06 μg/mL)/streptomycin (0.1 μg/mL) solution. All the above differences could be responsible for the higher vulnerability of cells to Glu cytotoxic action (EC_50_ 40 μM), and the protective effect of ED observed in Wu et al. study. In our model, where a higher concentration of Glu was used (EC_50_ 1000 μM), no protective effect of ED was detected. We observed that ED attenuated the cell damage induced by H_2_O_2_ in cortical neurons, but we did not find any protection by this compound in SH-SY5Y cells against the same cell-damaging factor under any of the tested experimental conditions. This is in contrast to Jami et al. [60] (2015) work, which demonstrated that ED (25 μM) attenuated cell damage induced by H_2_O_2_ in undifferentiated SH-SY5Y cells. The discrepancies between our and Jami’s data could be explained by using various concentrations and times of treatment with H_2_O_2_ (0.375 mM for 24 h vs. 2 mM for 8 h in our and Jami’s study, respectively), which could evoke different processes (apoptosis vs. necrosis).

Among tested compounds, CA demonstrated the highest neuroprotective effects against the H_2_O_2_-evoked cell damage in primary neuronal cell cultures as well as in UN- and RA-SH-SY5Y cells cultured in NB medium. This is in line with other reports showing the ability of CA (1–10 μM) to protect against cell damage induced by H_2_O_2_ in various cell types, including human neuroblastoma cells [35,49,61,62]. Moreover, CA was also protective in other oxidative stress-based neuronal cell damage models like those induced by 6-OHDA, paraquat, or dieldrin [37,49,63]. Moreover, our findings that CA was protective against the H_2_O_2_-induced cell damage only in UN- and RA-SH-SY5Y cells cultured in NB but not DMEM points to the importance of cell medium composition on the biological response of tested compounds, which should also be taken into account when comparing data with findings of other research groups [64]. Although some previous data showed that CA (1–10 μM) could protect neuronal cells against the Glu-induced cell damage in SH-SY5Y, HT-22 or PC12 cells, it was rather associated with the inhibition of oxytosis induced by Glu in these cells [34,43,65,66,67]. Our data demonstrating that CA is not protective against the Glu-mediated excitotoxicity in 8 days in vitro primary cortical neurons are in contrast to results obtained by Satoh et al. [65], who showed that CA (3 μM) alleviated the Glu-evoked cell damage in 21 days in vitro cortical neurons. It is not excluded that the neuroprotective effects of CA against the Glu-evoked excitotoxic damage are dependent on the developmental stage of the neurons (immature vs. mature ones). In our study, we also did not find any protection by CA in primary neurons against the OGD-evoked cell damage; however, this effect could be cell-type specific since previous reports showed that CA (1 μM) could attenuate the ischemia or hypoxia-evoked cell damage in PC12 cells which was connected with its antioxidative properties [68].

Regarding mechanisms of CA-mediated protection against H_2_O_2_-evoked cell damage, our results on ROS scavenging properties of CA are in line with previous findings [49,69]. However, a puzzling observation was that the inhibitory effect of CA on the H_2_O_2_-induced cortical neuronal damage was accompanied by ameliorating of reduction of mitochondrial membrane potential, but, unexpectedly, also with a moderate increase in caspase-3 activity. At least a previous study with SH-SY5Y cells showed that CA-mediated protection against H_2_O_2_ was associated, among others, with an increase in MMP and inhibition of caspase-3 [49]. Similar observations were made in the model of H_2_O_2_-evoked cell damage in HepG2 cells [35] and in 6-OHDA-evoked cell death in SH-SY5Y cells [63]. It is not excluded that this could be a cell-specific effect, and that could also be regulated by the developmental stage of the neuronal cells. Nevertheless, as evidenced by morphological analysis in our study, CA decreased the number of pyknotic nuclei, which is a hallmark of condensed or fragmented DNA evoked by apoptotic- and necrotic-like processes and protected both neuronal and glia cells against the H_2_O_2_-evoked cell death. It should be added that the importance of glia cells in CA-mediated protection was also highlighted in previous studies [65,70]. When taking into account the potential usefulness of CA in the treatment of neurodegenerative diseases [28,71], it is justified to consider it as an active component of nanodrugs, which could balance its unfavorable pharmacokinetic profile [12]. To this end, it has been reported that nanocarrier-packaged CA ameliorated glia-mediated neuroinflammation and improved cognitive function in an Alzheimer’s disease model [72]. 

## 3. Materials and Methods

### 3.1. Chemicals

Neurobasal A, Dulbecco’s Modified Eagle’s Medium (DMEM), FluoroBrite™ DMEM, supplement B27 (*w*/*o* antioxidants), fetal bovine serum (FBS), 0.25% trypsin/EDTA and penicillin/streptomycin were from Gibco (Life Technologies Ltd., Paisley, UK). The Cyto-toxicity Detection Kit and Cell Proliferation Reagent WST-1 were from Roche Diagnostics GmbH (Mannheim, Germany). Caspase-3 (Ac-DEVD-AMC) fluorogenic substrate and CM-H_2_DCFDA were obtained from Enzo Life Sciences (New York, NY, USA) and Molecular Probes (Life Technologies Corporation, Eugene, OR, USA), respectively. Edaravone, ebselen and carnosic acid were purchased from Selleck Chemicals LLC (Houston, TX, USA). All other reagents were from Sigma (Sigma-Aldrich Chemie GmbH, Taufkirchen, Germany). 

### 3.2. Primary Neuronal Cell Cultures

Neuronal cell cultures were prepared from cortices of mouse CD1 embryos (15/16 days of gestation) in accordance with the procedure described in detail previously [41]. The isolated cortical tissue was trypsinized (0.1% trypsin in PBS *w*/*o* Ca^2+^/Mg^2+^), the obtained cells were manually counted (Bürker chamber) and seeded on poly-L-ornithine (0.05 mg/mL)-covered plates at densities 6 × 10^4^ and 3 × 10^5^ cells per well in 96- and 24-well plates, respectively. The cells were cultured in Neurobasal A medium (minus phenol red, catalog No. 12349015) supplemented with 0.4% B27 (minus antioxidants, catalog No. 10889038) and antibiotics (0.06 μg/mL penicillin and 0.1 μg/mL streptomycin) with medium exchange every two days. For the first two days, the medium was additionally supplemented with 5% FBS to increase in culture the glia content. The cells were maintained at 37 °C in a humidified atmosphere containing 5% CO_2_ for eight days prior to experimentation with medium exchange every two days. Pregnant animals were obtained from Charles River Laboratories (Sulzfeld, Germany). The protocol for generating the primary neuronal cell cultures is in line with European Union (Directive 2010/63/EU, amended by Regulation (EU) 2019.1010) guidelines on the ethical use of animals and according to national regulations, it does not require the approval of the local ethics committee for animal research. All experiments were conducted according to the principles of the Three Rs, and all efforts were made to minimize the number of animals used and their suffering. 

### 3.3. SH-SY5Y Cell Culture 

The human SH-SY5Y neuroblastoma cell line was obtained from ATCC (CRL-2266, Manassas, VA, USA). The cells were grown in high glucose DMEM (Dulbecco’s Modified Eagle Medium) supplemented with 10% heat-inactivated FBS (fetal bovine serum) and 100 units/mL of penicillin and 100 μg/mL of streptomycin as described previously [41]. The cells were maintained at 37 °C in an atmosphere with saturated humidity containing 95% air and 5% CO_2_. Cells after reaching sufficient propagation rate (80% confluence in flasks) were detached from the surface with 0.05% trypsin/EDTA solution and manually counted in Bürker chamber. They were seeded at a density of 4 × 10^4^ or 2 × 10^4^ per well into 96-well plates for UN- and RA-SH-SY5Y, respectively. Differentiation of cells were performed by supplementation of cell culture medium with retinoic acid (RA, 10 µM) for six days with medium exchange every two days. One day before experiments, the culture medium for both cell phenotypes (UN-SH-SY5Y and RA-SH-SY5Y cells were replaced with two types of experimental medium: (i) neuroblastoma experimental medium (DMEM)—high glucose DMEM containing antibiotics and 1% FBS and (ii) neuronal experimental medium (NB)—Neurobasal A medium supplemented with 0.4% B27 (without antioxidants) and antibiotics (0.06 μg/mL penicillin and 0.1 μg/mL streptomycin) (NB). The cells were used for experiments between passages 4–17. Each new batch of SH-SY5Y, after rebanking, was regularly tested for putative Mycoplasma contamination with MycoBlueTM Mycoplasma Detector (Vazyme Biotech Co. Ltd., Nanjing, China). 

### 3.4. Cell Treatment

**Figure 10:**
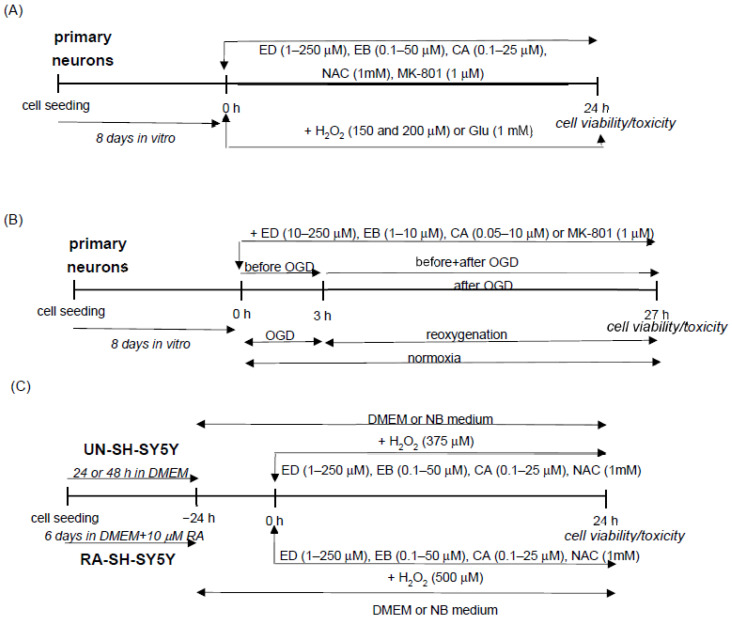
Cell treatment scheme for neuroprotection studies with edaravone (ED), ebselen (E and carnosic acid (CA) in: (**A**) primary neurons exposed to hydrogen peroxide (H_2_O_2_) or glutamate (Glu); (**B**) primary neurons exposed to oxygen-glucose deprivation (OGD); (**C**) undifferentiated (UN-), and retinoic acid-differentiated (RA-) SH-SY5Y cells exposed to H_2_O_2_.

First, primary neurons, UN-SH-SY5Y and RA-SH-SY5Y cells were treated with ED (100 and 250 μM), EB (5–50 μM), and CA (5–25 μM) for 24 h to assess the biosafety of the tested compounds. Next, all these cells were co-treated with ED (1–250 μM), EB (0.1–50 μM), CA, (0.1–25 μM) and H_2_O_2_ for 24 h. Moreover, we examined the effect of ED, EB, and CA in primary neurons against cell damage evoked by glutamate (Glu, 1 mM) or oxygen-glucose deprivation (OGD). As a positive control for the oxidative stress model, we used the anti-oxidant N-acetylcysteine (NAC, 1 mM), and for Glu and OGD models, we employed the NMDA receptor antagonist MK-801 (1 μM). The chosen concentrations of particular cytotoxic agents (H_2_O_2_ and Glu) and time of exposure (24 h; OGD 3 h followed by 24 h of reoxygenation) were optimized in our previous studies [41,73]. The schematic representation of cell treatment for neuroprotection screening is demonstrated in Figure 10. 

Stock solutions of ED (100 mM), EB (50 mM), and CA (100 mM) were prepared in DMSO, and their aliquots were kept at −80 °C. The final solutions of these compounds were pre-pared in a sterile distilled water/DMSO mixture and kept at −20 °C. Ac-DEVD-CHO (20 mM) stock solution was prepared in DMSO, and its final solution was prepared in distilled water. The H_2_O_2_ (100 mM) stock solution was prepared freshly from stabilized 30% hydrogen peroxide diluted to the final concentration in distilled water. The Glu (100 mM) stock solution was prepared immediately before use in 100 mM NaOH. The buffers for the OGD model were prepared according to the procedure described in our previous study [40]. Each experimental set of the control cultures were supplemented with the appropriate vehicle (sterile distilled water/DMSO mixture), and the solvent was present in cultures at a final concentration of 0.1%. All agents were added to the culture medium under light-limited conditions to avoid potential light-induced cytotoxicity.

### 3.5. Cell Viability Assays

We employed 3-[4,5-dimethylthiazol-2-yl]-2,5-diphenyltetrazolium bromide (MTT) assays for the assessment of cell viability in primary neuronal cell cultures as reported in our previous study [41]. To estimate cell viability in SH-SY5Y cells, we used the WST-1 reagent as described previously [74]. The data from 3–12 independent experiments with 3–5 replicates after normalization to the vehicle-treated cells (100%) are expressed as a percentage of the control ± SEM.

### 3.6. Cytotoxicity Assay

The Cytotoxicity detection kit (Roche) was used for measurement of released into culture media lactate dehydrogenase (LDH) as described in our previous study [41]. The data from 3–12 independent experiments with 3–5 replicates after normalization to the vehicle-treated cells (100%) are expressed as a percentage of the control ± SEM.

### 3.7. Immunofluorescence

In order to visualize cell viability data, primary neuronal cell cultures growing on poly-L-ornithine (0.05 mg/mL)-covered round cover glasses (diameter 12 mm) in 24-well plate format after treatment with CA (1–10 μM) and H_2_O_2_ (200 μM) were fixed with 4% paraformaldehyde and immunostained with neuronal (mouse anti-MAP-2, M9942 1:200, Sigma Aldrich) and astrocyte (GFAP, G9269 1:200, Sigma Aldrich) markers as described previously [41]. The samples, after overnight staining with primary antibodies (mouse ani-MAP2 and rabbit anti-GFAP) followed by 2 h labelling with relevant secondary anti-bodies (anti-mouse Alexa^®^488 and anti-rabbit Alexa^®^568, 1:250), were counterstained for 10 min with nuclear dye Hoechst 33342 and mounted in ProLong^®^Gold antifade reagent (Invitrogen, Waltham, MA, USA). The stained samples were imaged with the inverted fluorescence microscope (AxioObserver, Carl Zeiss, Jena, Germany) equipped with a black-white camera (Axio-CamMRm, Carl Zeiss). The excitation wavelengths were 470 nm for Alexa^®^488, 555 for Alexa^®^568 and 365 nm for Hoechst 33342. Under each fluorescence panel, four microphotographs were taken for all experimental groups in duplicates from three independent experiments. 

### 3.8. Measurement of Intracellular Reactive Oxygen Species (ROS) 

The intracellular ROS level was measured with 5-(and-6)-chloromethyl-2′,7′-dichlorodihydrofluorescein diacetate, acetyl ester (CM-H_2_DCFDA, Molecular Probe, USA) as described previously [40]. After washing with pre-warmed FluoroBrite™ DMEM, the cells were loaded with 5 μM CM-H_2_DCFDA (in FluoroBrite™ DMEM). Afterwards, the cells were treated with CA (0.1–50 μM), NAC (1 mM) and H_2_O_2_ (1 mM) and placed in an incubator for 1 h. Next, the cells were washed twice with pre-warmed FluoroBrite™ DMEM and the fluorescence was measured in a microplate multi-well reader (Infinite^®^ M200 PRO, Tecan, Switzerland) with excitation and emission wavelengths of 485 nm and 535 nm, respectively. The data were normalized to the vehicle-treated cells (100%) and presented as the mean ± SEM from four independent experiments with two to five replicates. 

### 3.9. Measurement of Mitochondrial Membrane Potential (MMP)

MMP was measured with the fluorescent probe tetramethylrhodamine ethyl ester (TMRE) as described previously [40]. The cells, after treatment with CA (0.1–10 μM) followed by 6 h of treatment with H_2_O_2_ (200 μM), were washed with pre-warmed FluoroBrite™ DMEM and loaded with 100 nM TMRE and located in an incubator for 20 min. After two washings with pre-warmed FluoroBrite™ DMEM, the fluorescence was measured in a multi-well microplate reader (Infinite^®^ M200 PRO, Tecan, Switzerland) with excitation and emission wavelengths of 540 nm and 595 nm, respectively. Data normalized to vehicle-treated cells (100%) were presented as the mean ± SEM from four independent experiments with three to five replicates.

### 3.10. Caspase-3 Activity Assay

The caspase-3 activity in primary neuronal cell cultures growing in 96-well format and treated with CA (1–10 μM) and H_2_O_2_ (200 μM) for 9 h was measured using the fluorogenic substrate Ac-DEVD-AMC (50 μM) as described previously [41]. Caspase-3 inhibitor, Ac-DEVD-CHO (20 μM), given 30 min before H_2_O_2_ exposure, was used as a positive control for the assay. The data were normalized to vehicle-treated cells (100%) and presented as the mean ± SEM from four independent experiments with three to five replicates.

### 3.11. Statistical Analysis

Data were analyzed using the Statistica 13 software (StatSoft Inc., Tulsa, OK, USA). The analysis of variance (one-way ANOVA) and post hoc Tukey’s test for multiple comparisons were used to show statistical significance with assumed *p* < 0.05. For comparison of two experimental groups was used *t* test with *p* < 0.05.

## 4. Conclusions

Our results indicate that the neuroprotective potential of CA, ED, and EB may be revealed in vitro only under specific conditions, with their rather narrow micromolar concentrations, relevant cellular model, type of toxic agent, and exposure time. Nevertheless, of the three compounds tested, CA displayed the most consistent neuroprotective effects (Table 2).

## Figures and Tables

**Table 1:** Neuroprotective effect of carnosic acid (CA) against the H_2_O_2_-induced cytotoxicity in UN- and RA-SH-SY5Y under various experimental mediums (DMEM vs. NB).

	UN-SH-SY5Y Cells		RA-SH-SY5Y Cells	
	DMEM	NB	DMEM	NB
control +veh	100 ± 0.0	100 ± 0.0	100 ± 0.0	100 ± 0.0
H_2_O_2_ + veh	359.14 ± 1.7 ***	699.82 ± 2.3 ***	245.01 ± 0.1 ***	482.32 ± 2.6 ***
H_2_O_2_ + CA 0.1	316.88 ± 20.7 ***	503.94 ± 20.3 ***^, #^	205.28 ± 8.1 ***	365.91 ± 10.8 ***^, ##^
H_2_O_2_ + CA 0.5	264.75 ± 36.2 **	458.69 ± 32.4 ***^, ##^	204.91 ± 17.3 ***	348.60 ± 12.4 ***^, ###^
H_2_O_2_ + CA 1	257.14 ± 27.2 ***^, #^	384.59 ± 40.6 ***^, ###^	193.69 ± 9.5 ***	324.65 ± 16.2 ***^, ###^
H_2_O_2_ + CA 5	232.57 ± 32.6 **^, ##^	296.23 ± 63.9 *^, ###^	196.07 ± 12.5 ***	199.67 ± 9.6 *^, ###^
H_2_O_2_ + CA 10	317.90 ± 29.2 ***	400.47 ± 54.6 ***^, ###^	207.87 ± 20.8 ***	226.16 ± 41.9 ***^, ###^
n	4–7	6–7	4–5	4

The UN- and RA-SH-SY5Y cells growing overnight in neuroblastoma (DMEM), or neuronal (NB) experimental medium were treated either with vehicle or with CA (0.1–10 μM) in combination with H_2_O_2_ (375 and 500 μM for UN- and RA-SH-SY5Y cells, respectively) for 24 h. Cell cytotoxicity was measured by LDH release assay. Data after normalization to vehicle-treated cells (100%) are presented as a mean ± SEM. The number of independent experiments (n) is indicated in separate rows. * p < 0.05, ** p < 0.01 and *** *p* < 0.001 vs. vehicle-treated cells; # *p* < 0.05, ## *p* < 0.01 and ### *p* < 0.001 vs. H_2_O_2_- treated cells.

**Table 2:** Summary of neuroprotective potency of edaravone, ebselen, and carnosic acid in various neuronal models.

			UN-SH-SY5Y		RA-SH-SY5Y	
Cell Damage Model		Primary Cortical Neurons	DMEM	NB	DMEM	NB
	ED	+	+/−	+/−	+/−	+/−
H_2_O_2_	EB	+	−	+/−	−	+/−
	CA	+++	+/−	+++	+/−	+++
	ED	+/−				
Glu	EB	− −	n.d.	n.d.	n.d.	n.d
	CA	+/−				
	ED	+/−				
OGD	EB	− −	n.d.	n.d.	n.d.	n.d.
	CA	−				

“+”—protective; “+/−”—no protective; “−”—detrimental; CA—carnosic acid; EB—ebselen; ED—edaravone; Glu—glutamate; H_2_O_2_—hydrogen peroxide; n.d.—not determined; OGD—oxygen-glucose deprivation; UN-SH-SY5Y—undifferentiated SH-SY5Y cells; RA-SH-SY5Y—retinoic acid-differentiated SH-SY5Y cells.

## Data Availability

The raw data that support the findings of this study are available from the first/corresponding author [D.J.] upon reasonable request.

## Supplementary Materials

The following supporting information can be downloaded at: https://www.mdpi.com/article/10.3390/molecules29010119/s1, Figure S1: Neuroprotective effects of edaravone (ED, A,B) and ebselen (EB, C,D) against the glutamate (Glu)-induced cell damage in primary neuronal cell cultures; Figure S2: Neuroprotective effect of edaravone (ED, A,B) and ebselen (EB, C,D) against the oxygen-glucose deprivation (OGD)-induced cell damage in primary neuronal cell cultures; Figure S3: Neuroprotective effect of edaravone (ED) against the H_2_O_2_-induced cell damage in UN- and RA-SH-SY5Y under various experimental medium (DMEM vs. NB). Figure S4: Neuroprotective effect of ebselen (EB) against the H_2_O_2_-induced cell damage in UN- and RA-SH-SY5Y under various experimental medium (DMEM vs. NB).

## Abbreviations

6-OHDA—6-hydroxydopamine; CA—carnosic acid; EB—ebselen; ED—edaravone; Glu—glutamate; H_2_O_2_—hydrogen peroxide; LDH—lactate dehydrogenase; MMP—mitochondrial membrane potential; MTT—3-[4,5-dimethylthiazol-2-yl]-2,5-diphenyltetrazolium bromide; OGD—oxygen-glucose deprivation; RA—retinoic acid; RA-SH-SY5Y—retinoic acid-differentiated SH-SY5Y cells; RNS—reactive nitrogen species; ROS—reactive oxygen species; TMRE—tetramethylrhodamine ethyl ester; UN-SH-SY5Y—undifferentiated SH-SY5Y cells.
