# Supplementary material for: Carnosic Acid Shows Higher Neuroprotective Efficiency than Edaravone or Ebselen in In Vitro Models of Neuronal Cell Damage
Source: Molecules. 2023 Dec 24;29(1):119. doi: 10.3390/molecules29010119 (PMC10779571; doi:10.3390/molecules29010119)
Supplement: Supplementary file 1 [file molecules-29-00119-s001.zip › molecules-2749816-supplementary.pdf]

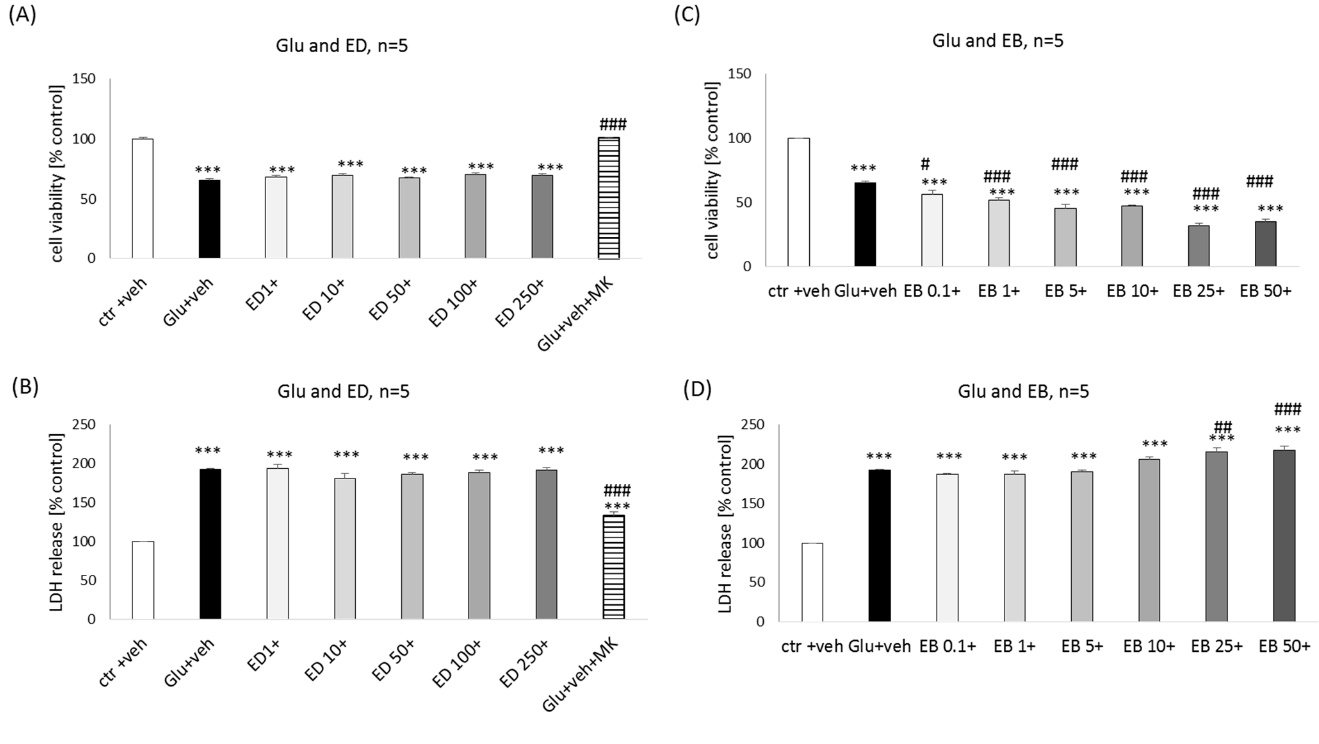

Figure S1. Neuroprotective effects of edaravone (ED, **A–B**) and ebselen (EB, **C–D**) against the glutamate (Glu)-induced cell damage in primary neuronal cell cultures. The eight days in vitro cortical neurons were treated either with vehicle or with ED (1–250 µM) or EB (0.1–50 µM) in combination with Glu (1 mM) for 24 h. NMDA receptor antagonist MK-801 (1 µM) was used as a positive control to the model. Cell viability (**A, C**) and cytotoxicity (**B, D**) were measured by MTT reduction and LDH release assays, respectively. The data were normalized to vehicle-treated cells and presented as the mean ± SEM. The number of independent experiments (*n*) is indicated in each graph. *** $p < 0.001$ vs. vehicle-treated cell; # $p < 0.05$, ## $p < 0.01$ and ### $p < 0.001$ vs. Glu-treated cells.

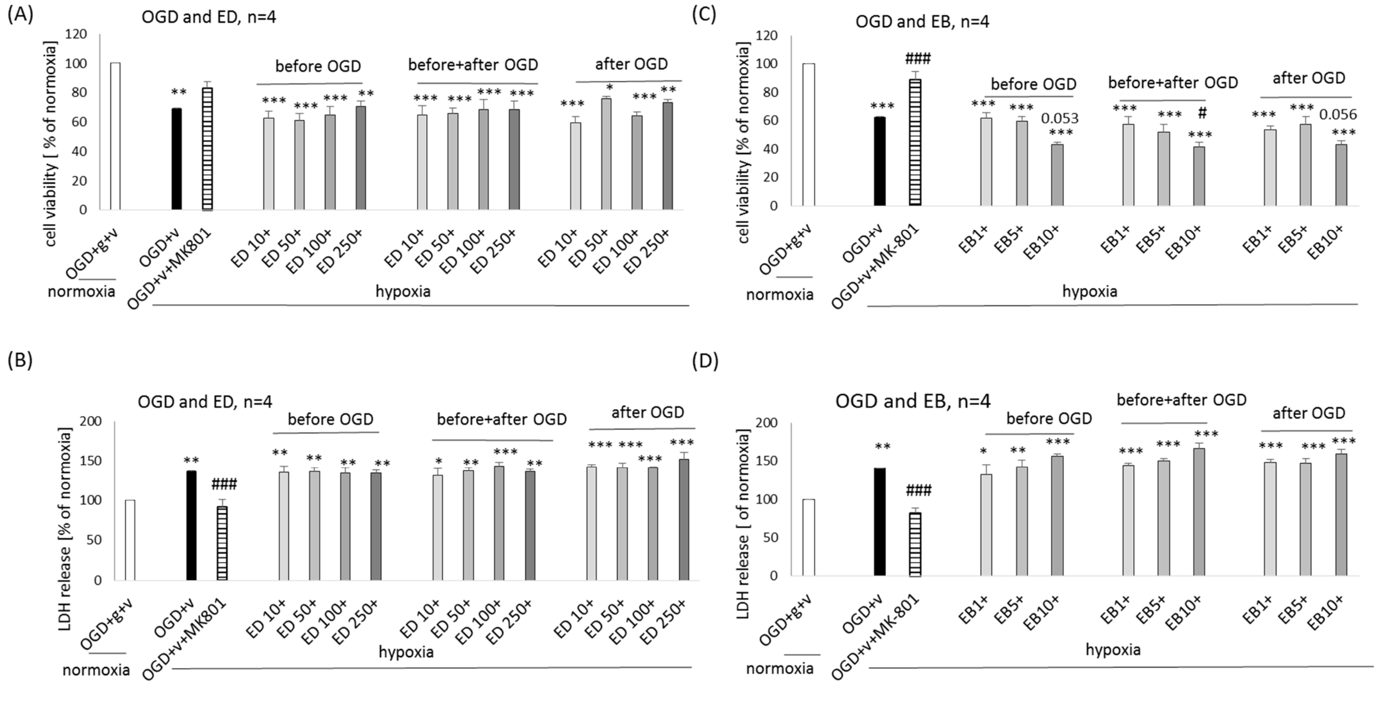

Figure S2. Neuroprotective effect of edaravone (ED, **A–B**) and ebselen (EB, **C–D**) against the oxygen-glucose deprivation (OGD)-induced cell damage in primary neuronal cell cultures. The eight days in vitro cortical neurons were treated either with vehicle or with ED (10–250 µM) or EB (1–10 µM) under three schedules (before OGD, before+after OGD, after OGD) combined with 3 h OGD procedure and 24 h of reoxygentaion period. NMDA receptor antagonist, MK-801 (1 µM) given before+after OGD was used as a positive control to the model. Cell viability (**A, C**) and cytotoxicity (**B, D**) were measured by MTT reduction and LDH release assays, respectively. The data were normalized to vehicle-treated cells and presented as the mean ± SEM. The number of independent experiments (*n*) is indicated in each graph. * $p < 0.05$, ** $p < 0.01$ and *** $p < 0.001$ vs. vehicle-treated cell; ### $p < 0.001$ vs. OGD-treated cells.

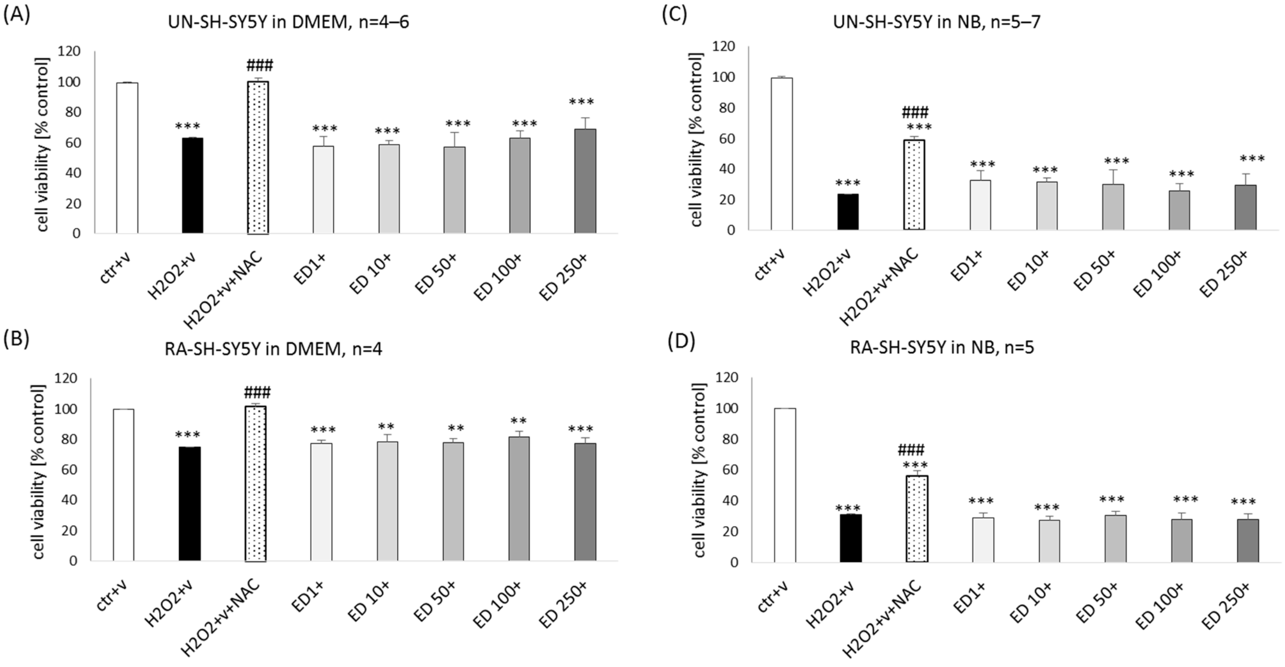

Figure S3. Neuroprotective effect of edaravone (ED) against the H_2O_2-induced cell damage in UN- and RA-SH-SY5Y under various experimental mediums (DMEM vs. NB). The UN- and RA-SH-SY5Y cells growing overnight in neuroblastoma (DMEM) or neuronal (NB) experimental medium were treated either with vehicle or with ED (1–250 µM) in combination with H_2O_2 (375 µM and 500 µM for UN- and RA-SH-SY5Y cells, respectively) for 24 h. An antioxidant N-acetyl-cysteine (NAC, 1 mM) was used as a positive control of the model. Cell viability was measured by WST-1 assay. Data after normalization to vehicle-treated cells (100%) are presented as a mean ± SEM. The number of independent experiments (*n*) is indicated in each graph. ** $p < 0.01$ and *** $p < 0.001$ vs. vehicle-treated cells; ### $p < 0.01$ vs. H_2O_2- treated cells.

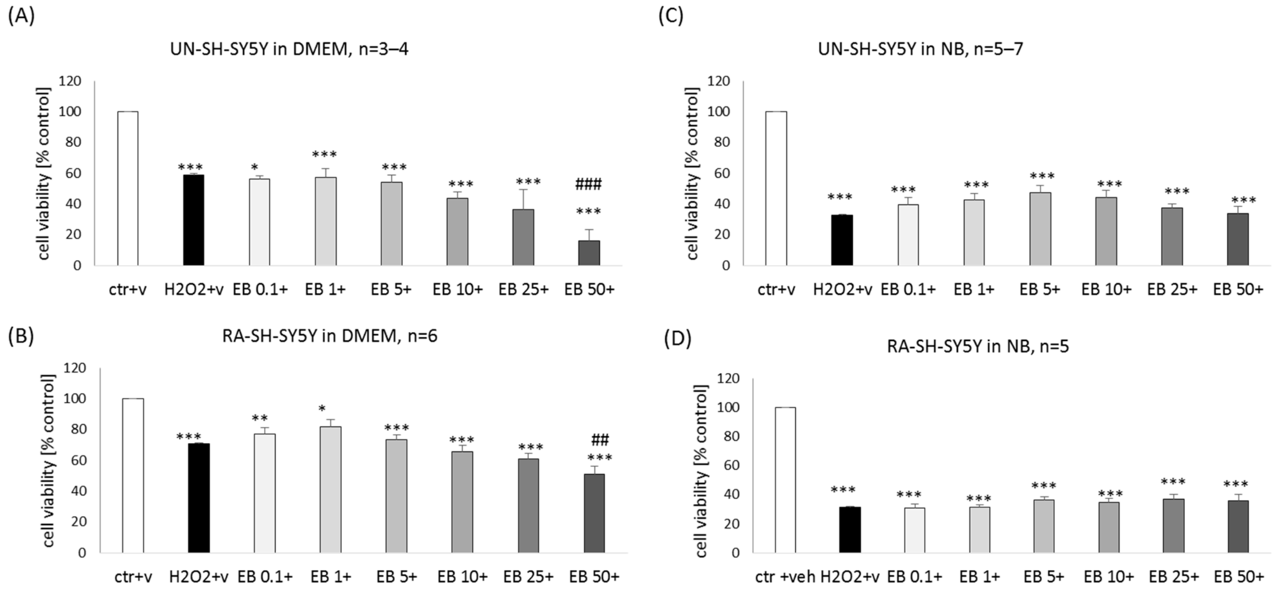

Figure S4. Neuroprotective effect of ebselen (EB) against the H_2O_2-induced cell damage in UN- and RA-SH-SY5Y under various experimental mediums (DMEM vs. NB). The cells growing overnight in neuroblastoma (DMEM) or neuronal (NB) experimental medium were treated either with vehicle or with EB (0.1–50 µM) in combination with H_2O_2 (0.375 and 0.5 mM for UN- and RA-SH-SY5Y cells, respectively) for 24 h. Cell viability was measured by WST-1 assay. Data after normalization to vehicle-treated cells (100%) are presented as a mean ± SEM. The number of independent experiments (*n*) is indicated in each graph. * p <0.05, ** p < 0.01 and *** p < 0.001 vs. vehicle-treated cells; ## p <0.05 and ### p < 0.001 vs. H_2O_2- treated cells.
